# Supplementary material for: RNA Sequencing (RNA-Seq) Reveals Extremely Low Levels of Reticulocyte-Derived Globin Gene Transcripts in Peripheral Blood From Horses (Equus caballus) and Cattle (Bos taurus)
Source: Front Genet. 2018 Aug 14;9:278. doi: 10.3389/fgene.2018.00278 (PMC6102425; doi:10.3389/fgene.2018.00278)
Supplement: Supplementary file 3 [file Table_3.docx]

Supplementary Material

RNA sequencing (RNA-seq) reveals extremely low levels of reticulocyte-derived globin gene transcripts in peripheral blood from horses (*Equus caballus*) and cattle (*Bos taurus*)

**Carolina N. Correia^1^, Kirsten E. McLoughlin^1^, Nicolas C. Nalpas^1, †^, David A. Magee^1^, John A. Browne^1^, Kevin Rue-Albrecht^1, §^, Stephen V. Gordon^2, 3^, David E. MacHugh^1, 3^***

^1^ Animal Genomics Laboratory, UCD School of Agriculture and Food Science, UCD College of Health and Agricultural Sciences, University College Dublin, Belfield, Dublin, D04 V1W8, Ireland.

^2^ UCD School of Veterinary Medicine, UCD College of Health and Agricultural Sciences, University College Dublin, Belfield, Dublin, D04 V1W8, Ireland.

^3^ UCD Conway Institute of Biomolecular and Biomedical Research, University College Dublin, Belfield, Dublin, D04 V1W8, Ireland.

^†^ Current address: Quantitative Proteomics and Proteome Centre Tübingen, Interfaculty Institute for Cell Biology, University of Tübingen, Tübingen, 72076, Germany.

^§^ Current address: Kennedy Institute of Rheumatology, University of Oxford, Oxford OX3 7FY, United Kingdom.

*** Correspondence:** David E. MacHugh, Animal Genomics Laboratory, UCD College of Health and Agricultural Sciences, University College Dublin, Belfield, Dublin D04 V1W8, Ireland.

Email: [david.machugh@ucd.ie](mailto:david.machugh@ucd.ie)

# Supplementary Tables

**Supplementary Table 1:** Human, porcine, equine, and bovine sample and RNA-seq information (Excel file – **Supp_table_01.xlsx**).

**Supplementary Table 2:** Bovine sample information with total RNA quality and quantity data and RNA-seq library pool and barcode index information (Excel file – **Supp_table_02.xlsx**).

**Supplementary Table 3** (see overleaf).

**Supplementary Table 4:** Complete RNA-seq filtering/trimming statistics (Excel file – **Supp_table_04.xlsx**).

**Supplementary Table 5:** Complete RNA-seq mapping statistics (Excel file – **Supp_table_05.xlsx**).

**Supplementary Table 6:** Proportion of haemoglobin gene-level TPM per sample. (Excel file – **Supp_table_06.xlsx**).

**Supplementary Table 3:** Detailed information about the reference transcriptomes used in the present work.

| **Organism** | **Annotation release** | **Genome assembly** | **Source of reference transcriptome*** | **Date downloaded** | **No. of features**** |
| --- | --- | --- | --- | --- | --- |
| *Homo sapiens* | NCBI *Homo sapiens* Annotation Release 109 (March 2018)^1^ | GRCh38.p12 (GCF_000001405.38, December 2017)^2^ | [www.ncbi.nlm.nih.gov/genome/annotation_euk/Homo_sapiens/109/](http://www.ncbi.nlm.nih.gov/genome/annotation_euk/Homo_sapiens/109/)  ftp://ftp.ncbi.nih.gov/genomes/Homo_sapiens/RNA/rna.fa.gz | May 2018 | 159,998 |
| *Sus scrofa* | NCBI *Sus scrofa* Annotation Release 106 (May 2017)^1^ | Sscrofa11.1 (GCF_000003025.6, February 2017)^3, 4^ | [www.ncbi.nlm.nih.gov/genome/annotation_euk/Sus_scrofa/106/](http://www.ncbi.nlm.nih.gov/genome/annotation_euk/Sus_scrofa/106/)  [ftp.ncbi.nlm.nih.gov/genomes/Sus_scrofa/RNA/rna.fa.gz](ftp://ftp.ncbi.nlm.nih.gov/genomes/Sus_scrofa/RNA/rna.fa.gz) | August 2017 | 77,689 |
| *Equus caballus* | NCBI *Equus caballus* Annotation Release 103 (January 2018)^1^ | EquCab3 (GCF_002863925.1, May 2018)^5^ | [www.ncbi.nlm.nih.gov/genome/annotation_euk/Equus_caballus/103/](http://www.ncbi.nlm.nih.gov/genome/annotation_euk/Equus_caballus/103/)  ftp://ftp.ncbi.nlm.nih.gov/genomes/Equus_caballus/RNA/rna.fa.gz | May 2018 | 76,567 |
| *Bos taurus* | NCBI *Bos taurus* Annotation Release 106 (May 2018)^1^ | ARS-UCD1.2 (GCF_002263795.1, April 2018) ^6^ | www.ncbi.nlm.nih.gov/genome/annotation_euk/Bos_taurus/106/  ftp://ftp.ncbi.nih.gov/genomes/Bos_taurus/RNA/rna.fa.gz | May 2018 | 76,341 |

* NCBI FTP links to reference transcriptomes always direct the user to the most recent file available. Past annotation releases can be found on the NCBI FTP site ‘ARCHIVE’ directory.

** Known RefSeq (accession prefixes NM_ and NR_) and model RefSeq (XM_ and XR_) mRNAs, non-coding RNAs, and pseudo transcripts. For more details, see: <https://www.ncbi.nlm.nih.gov/refseq/about/>

^1^ O'Leary N.A., *et al.* (2016) Reference sequence (RefSeq) database at NCBI: current status, taxonomic expansion, and functional annotation. *Nucleic Acids Res.* **44**, D733-45.
^2^ Lander E.S., *et al.* (2001) Initial sequencing and analysis of the human genome. *Nature* **409,** 860-921.
^3^ Uenishi H., *et al.* (2012) Large-scale sequencing based on full-length-enriched cDNA libraries in pigs: contribution to annotation of the pig genome draft sequence. *BMC Genomics* **13**, 581.
^4^ Groenen M.A., *et al.* (2012) Analyses of pig genomes provide insight into porcine demography and evolution. *Nature* **491**, 393-8.
^5^ Kalbfleisch T.S., *et al.* (2018) EquCab3, an Updated Reference Genome for the Domestic Horse. *bioRxiv*, 306928.

^6^ Rosen B.D., *et al.* (2018) Modernizing the Bovine Reference Genome Assembly. *Proceedings of the World Congress on Genetics Applied to Livestock Production*, **Molecular Genetics 3**, 802.
